# Structural analysis of the boronic acid β-lactamase inhibitor vaborbactam binding to *Pseudomonas aeruginosa* penicillin-binding protein 3

**DOI:** 10.1371/journal.pone.0258359

**Published:** 2021-10-15

**Authors:** Vijay Kumar, Samantha L. Viviani, Jeeda Ismail, Shreya Agarwal, Robert A. Bonomo, Focco van den Akker

**Affiliations:** 1 Department of Biochemistry, Case Western Reserve University, Cleveland, Ohio, United States of America; 2 Louis Stokes Cleveland Veteran’s Affairs Medical Center Research Service, Cleveland, Ohio, United States of America; 3 Department of Medicine, Case Western Reserve University, Cleveland, Ohio, United States of America; 4 Department of Molecular Biology and Microbiology, Case Western Reserve University, Cleveland, Ohio, United States of America; 5 Department of Pharmacology, Case Western Reserve University, Cleveland, Ohio, United States of America; 6 Department of Proteomics and Bioinformatics, Case Western Reserve University, Cleveland, Ohio, United States of America; 7 VA Center for Antimicrobial Resistance and Epidemiology (Case VA CARES), Case Western Reserve University, Cleveland, Ohio, United States of America; University of Cambridge, UNITED KINGDOM

## Abstract

Antimicrobial resistance (AMR) mediated by β-lactamases is the major and leading cause of resistance to penicillins and cephalosporins among Gram-negative bacteria. β-Lactamases, periplasmic enzymes that are widely distributed in the bacterial world, protect penicillin-binding proteins (PBPs), the major cell wall synthesizing enzymes, from inactivation by β-lactam antibiotics. Developing novel PBP inhibitors with a non-β-lactam scaffold could potentially evade this resistance mechanism. Based on the structural similarities between the evolutionary related serine β-lactamases and PBPs, we investigated whether the potent β-lactamase inhibitor, vaborbactam, could also form an acyl-enzyme complex with *Pseudomonas aeruginosa* PBP3. We found that this cyclic boronate, vaborbactam, inhibited PBP3 (IC_50_ of 262 μM), and its binding to PBP3 increased the protein thermal stability by about 2°C. Crystallographic analysis of the PBP3:vaborbactam complex reveals that vaborbactam forms a covalent bond with the catalytic S294. The amide moiety of vaborbactam hydrogen bonds with N351 and the backbone oxygen of T487. The carboxyl group of vaborbactam hydrogen bonds with T487, S485, and S349. The thiophene ring and cyclic boronate ring of vaborbactam form hydrophobic interactions, including with V333 and Y503. The active site of the vaborbactam-bound PBP3 harbors the often observed ligand-induced formation of the aromatic wall and hydrophobic bridge, yet the residues involved in this wall and bridge display much higher temperature factors compared to PBP3 structures bound to high-affinity β-lactams. These insights could form the basis for developing more potent novel cyclic boronate-based PBP inhibitors to inhibit these targets and overcome β-lactamases-mediated resistance mechanisms.

## Introduction

Antimicrobial resistance (AMR) is a global health concern. The largest class of antibiotics are penicillin-binding protein (PBP)-targeting β-lactam antibiotics such as penicillin and members of the cephalosporin and carbapenem classes. Unfortunately, bacterial pathogens possess a number of β-lactam resistance mechanisms of which the expression of β-lactam-degrading enzymes, β-lactamases, are among the main mechanisms [1]. Pathogens harbor additional β-lactam antibiotic resistance mechanisms. For example, *P*. *aeruginosa* already has a basal increased resistance to antibiotics due to the inducible expression of the AmpC β-lactamase (*bla*_PDC_), the expression of efflux pumps, and reduced permeability of the outer membrane [2–6]. Additional acquired β-lactam resistance is mediated via AmpR mutations that increase AmpC/PDC β-lactamase expression [7], mutations in PBP3 [4, 7], efflux pump mutations [7], presence of other β-lactamases such as VIM-2 and GES [7], and downregulation or mutations in OprD porins [4, 7, 8] affecting carbapenem uptake. More recently, a deficiency in the PiuA iron transporter for the siderophore cephalosporin cefiderocol [9] has also been described. Unfortunately, ceftolozane-tazobactam that was recently introduced to treat *P*. *aeruginosa* infections has lost some efficacy; resistance has emerged as a result of horizontal transfer of β-lactamases [4, 7] or substitutions in PDC β-lactamase [10], allowing for this disturbingly rapid emergence of this resistant phenotype.

Nevertheless, PBPs are still valid antibacterial drug targets as they are responsible for the vital crosslinking of peptidoglycan strands. The β-lactams’ ability to inactivate these enzymes has shortcomings in terms of resistance development largely due to β-lactamases. One approach to overcome β-lactamases is combination therapy with a β-lactamase inhibitor. Unfortunately, resistance to this therapeutic strategy is also observed for many β-lactam:β-lactamase inhibitor combinations [11]. An alternative approach is to develop non-β-lactam PBP inhibitors to avoid degradation by β-lactamases. Such compounds include diazabicyclooctane (DBOs) as PBP inhibitors/β-lactam “enhancers” [12–18], γ-lactams [19–22], and boronic acid transition state analogs [23–27]. Ideas for such different scaffolds often arose from β-lactamase inhibitor development efforts as serine β-lactamases and PBPs are evolutionarily related and share the same catalytic serine, other active site residues, and mechanism. Some of these compounds are dual-target inhibitors and can covalently bind both PBPs and β-lactamases, such as the DBOs zidebactam and WCK 5153 [15, 18]. Remarkably, because of their PBP-inhibiting abilities, these latter compounds have antibiotic properties themselves [15]. Compared to the DBOs, boronic acid transition state analogs have lagged somewhat in their development as dual-target or PBP inhibitors, although great strides have been made towards their inhibition of β-lactamases.

**Fig 1:**
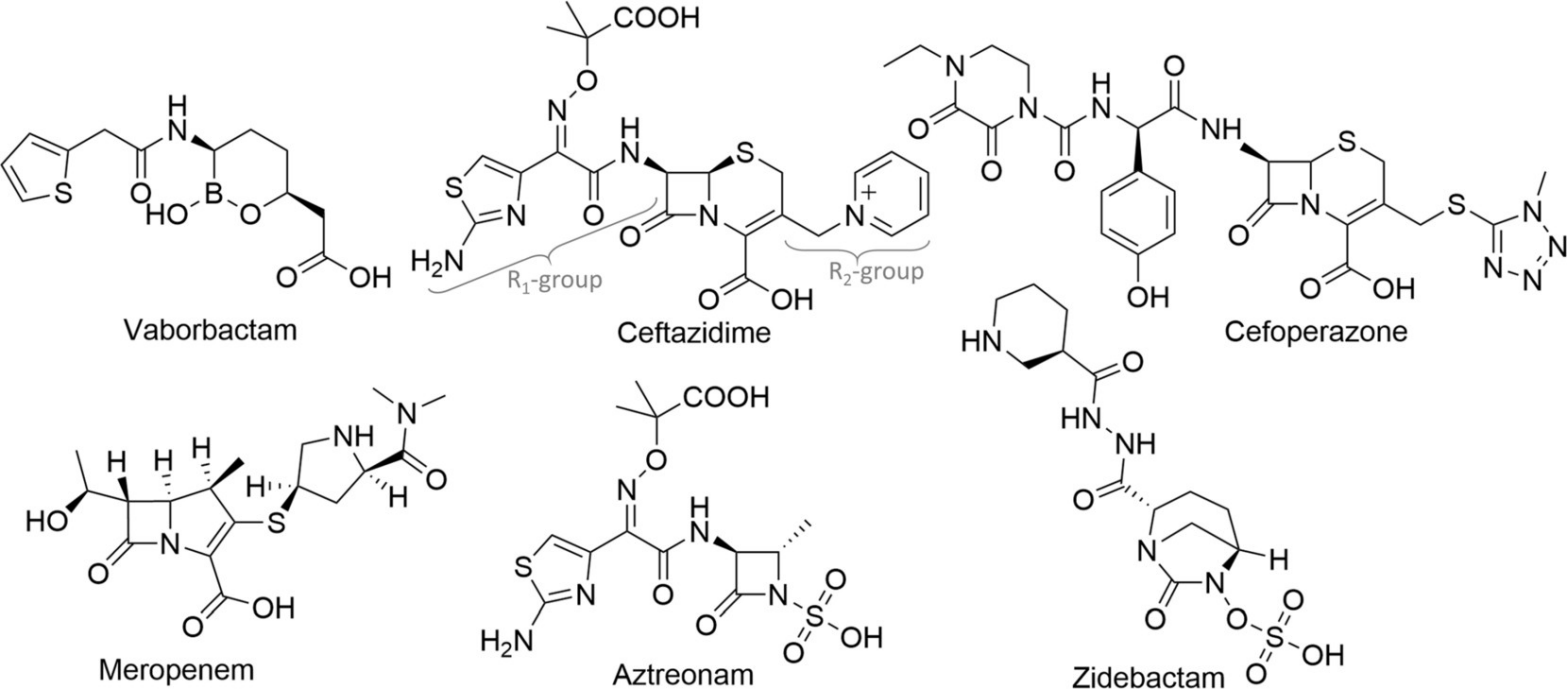
Chemical structures of vaborbactam and β-lactams, a monobactam and a DBO inhibitor. Vaborbactam, ceftazidime, cefoperazone, meropenem, aztreonam, and zidebactam are represented. The R_1_-and R_2_-groups for ceftazidime are indicated.

Vaborbactam is an FDA-approved cyclic boronate β-lactamase inhibitor (Fig 1) and is combined with meropenem [28]; the pre-clinical bicyclic boronic acids QPX7728 [24] and taniborbactam/VNRX-5133 [29] are very promising in that they inhibit both serine- and metallo- β-lactamases. To test whether the β-lactamase inhibitor vaborbactam can extend its inhibition ability to a homologous PBP, we probed the binding of vaborbactam to *P*. *aeruginosa* PBP3. We carried out crystallographic, biochemical, biophysical, and molecular dynamics (MD) simulations of the vaborbactam:PBP3 complex. Our gained insights could lead to further development of boronic acids as non-β-lactam PBP inhibitors or even “dual-target” inhibitors.

## Materials and methods

### Bocillin™ competition assay

The *P*. *aeruginosa* PBP3 protein was expressed and purified as previously described [19, 30, 31]. The Bocillin™ competition assay was done in a similar manner as described previously [19]. PBP3 (0.19 μM) was incubated with varying concentrations of vaborbactam in 10 mM sodium phosphate pH 7.4, 150 mM NaCl for 15 minutes at room temperature before adding 1 μM Bocillin™ and continued the incubation for 15 min at 37°C before boiling the samples and subsequent SDS PAGE fluorescence analysis of the PBP3:Bocillin™ complex at λ = 488 nm wavelength using a Bio-rad ChemiDoc MP Imaging System. The data were analyzed, and IC_50_ determinations were done using GraphPad Prism 9 (GraphPad Software, La Jolla, California USA).

### Differential Scanning Fluorimetry (DSF)/thermal shift assay

The DSF assay was done as previously described [19, 31]. PBP3 at 3 μM in 100 mM sodium phosphate pH 7.4, 100 mM NaCl buffer with 12x SYPRO orange with different vaborbactam concentrations. The experiments were carried out in duplicate. The temperature was ramped from 25.0–89.5°C, and the fluorescence was measured using a CFX96 Touch Real-Time PCR Detection System (Bio-Rad). The vaborbactam stock solution was prepared in DMSO; the DMSO concentrations were kept constant at 4% in the DSF experiments with varying vaborbactam concentrations.

### Structure determination

Crystallization of apo PBP3 was carried out as previously described [19, 30, 31]. Vaborbactam was soaked into a PBP3 crystal for 1 hour at 5 mM concentration in mother liquor before freezing the crystal in liquid nitrogen for data collection (no additional cryoprotectant was used). X-ray diffraction data were collected at the NSLS-II AMX beamline and processed to 2.2 Å resolution using XDS [32] (Table 1). The structure was solved via molecular replacement using PHASER [33] with the *P*. *aeruginosa* PBP3 ceftazidime complex protein coordinates [30] as the search model. Crystallographic refinement and model building were done using REFMAC5 [34] and COOT [35], respectively. Refinement parameter files for vaborbactam were generated using AceDRG [36]. The final PBP3 model contains residues 57–190, 213–490, and 501–561; one covalently bound vaborbactam molecule; and 81 water molecules (see Table 1 for additional refinement statistics). The protein coordinates and structure factors have been deposited at the Protein Data Bank (PDB id = 7LY1).

**Table 1:** X-ray diffraction data collection and crystallographic refinement statistics for the *P*. *aeruginosa* PBP3 complex with vaborbactam.

Wavelength (Å)	0.92012
Resolution range (Å)	29.49–2.20
Space group	P2_1_2_1_2_1_
Unit cell (Å, °)	68.10 83.50 88.42 90 90 90
Total reflections	346,804
Unique reflections	26,281 (1,864)^a^
Multiplicity	13.2 (11.6)^a^
Completeness (%)	99.8 (97.4)^a^
Mean I/sigma (I)	15.2 (2.8)^a^
CC_1/2_	0.997 (0.801)^a^
R-merge (%)	0.112 (0.836)^a^
Resolution refinement (Å)	27.81–2.20
Reflections used in refinement	24,839
Reflections used for R-free	1,393
R-work	0.199
R-free	0.241
Number of non-hydrogen atoms	3,715
Macromolecules	3,608
Ligand	26
Solvent	81
Protein residues	473
RMS(bonds, Å)	0.013
RMS(angles, °)	1.70
Ramachandran favored (%)	97.0
Ramachandran allowed (%)	3.0
Ramachandran outliers (%)	0.0
Average B-factor protein (Å^2^)	52.0
Average B-factor ligand (Å^2^)	63.9
Average B-factor solvent (Å^2^)	47.6

^a^Statistics for the highest-resolution shell are shown in parentheses.

### Molecular dynamics simulations

The coordinates of the PBP3-vaborbactam structure were used as starting models for MD simulation. The starting model included the crystallographically determined water molecules. Hydrogen atoms were added to the model using the Schrodinger Maestro protein preparation step. The PBP3 complex was placed in a box of explicit SPC water molecules with a 10 Å buffer in each direction. NaCl ions were added to neutralize the overall charge of the PBP3 complex, and additional salt (NaCl) ions were added to an overall ionic strength of 0.15 M NaCl, close to physiological NaCl concentration. The model was minimized and subsequently relaxed using default Desmond protocols prior to the 100 ns simulation step (at 310 K). The Desmond software in Schrödinger 2020–4 (Schrödinger, LLC, New York, NY) version was used, and the default OPLS3e force field [37] was selected.

## Results and discussion

### Affinity and thermal shift analysis of vaborbactam binding to PBP3

**Fig 2:**
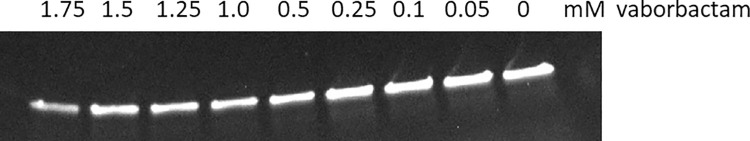
Bocillin™ competition assay probing inhibition of *P*. *aeruginosa* PBP3 by vaborbactam. SDS PAGE gel fluorescence scan of PBP3 reacted with the fluorescent Bocillin^TM^ reporter β-lactam in the presence of varying concentrations of the vaborbactam inhibitor.

To measure vaborbactam’s ability to inhibit *P*. *aeruginosa* PBP3, we carried out the Bocillin™ competition assay (Fig 2). The determined IC_50_ from this competition assay was 262 μM (95% CI: 142–487 μM). This IC_50_, corresponding to 78 μg/ml, is much higher than those of PBP3-targeting inhibitors such as the β-lactams ceftazidime, meropenem, and monobactam aztreonam with IC_50_ values 0.1, 0.08, and 0.03 μg/ml, respectively [30, 38, 39] (Fig 1). Note that the vaborbactam inhibition of PBP3 is anticipated to be reversible, in a similar manner to the inhibition of related β-lactamases [40], whereas inhibition of PBPs by β-lactams is also covalent yet with slow off-rates and turnover [41].

**Fig 3:**
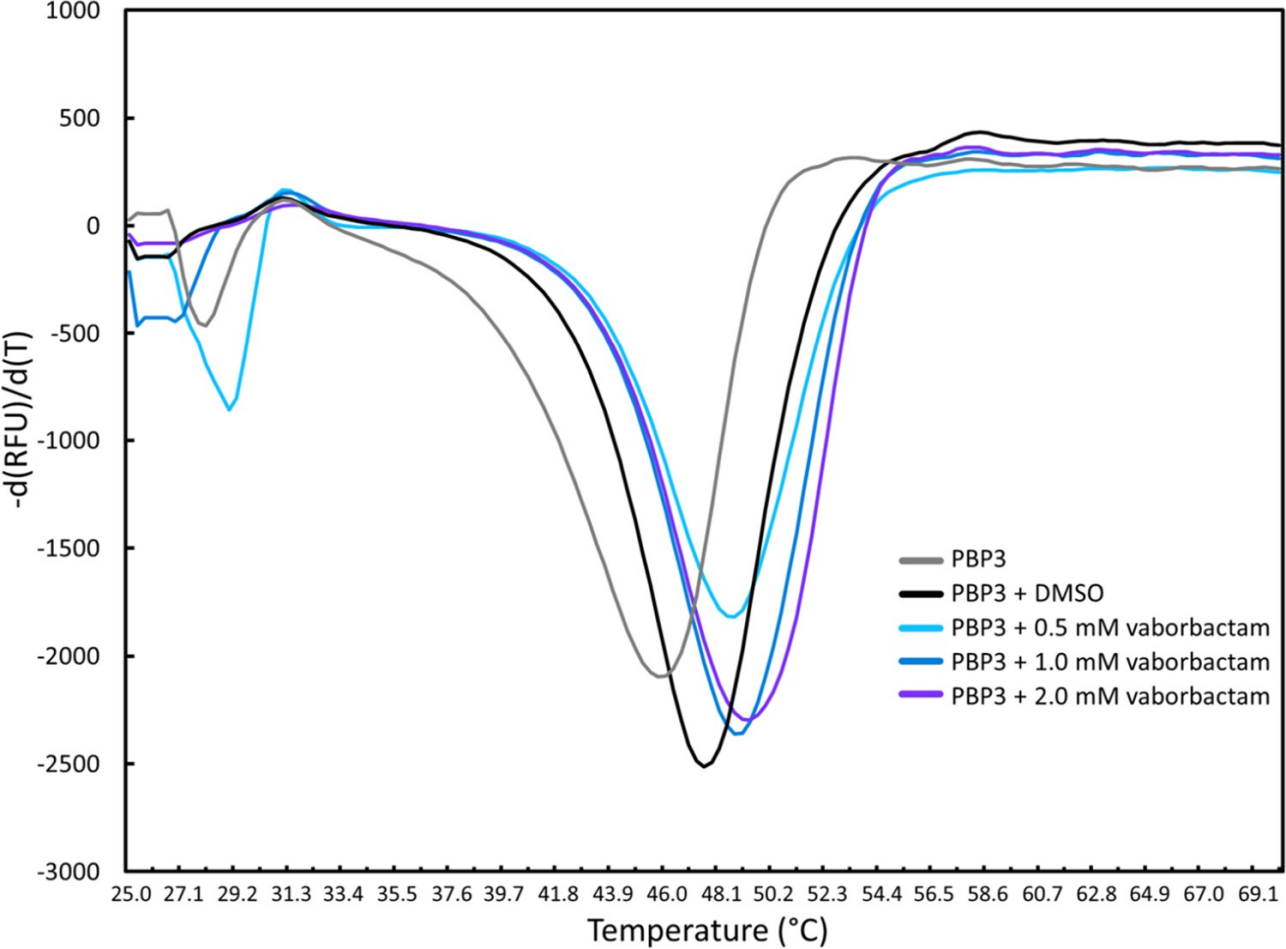
Differential Scanning Fluorimetry (DSF)/thermal shift assay probing protein stabilization of *P*. *aeruginosa* PBP3 by vaborbactam. The rate of change in SYPRO orange fluorescence is plotted versus temperature for PBP3 in the presence of varying concentrations of vaborbactam. Duplicate measured T_m_ values are as follows: PBP3 (with DMSO): 47.2 and 47.8°C; PBP3 with 0.5 mM vaborbactam (49.0 and 48.7°C), with 1.0 mM vaborbactam (49.0 and 49.0°C), and 2 mM vaborbactam (49.6 and 49.9°C).

Thermal shift/differential scanning fluorimetry (DSF) is a useful tool to probe the ligand’s effect on protein stability as has been previously carried out for a variety *P*. *aeruginosa* PBP3 inhibitors [18, 30, 31, 42]. Using DSF, vaborbactam binding to PBP3 was found to increase the thermal stability of PBP3: the melting temperature (*T*_m_) of PBP3 increased from 47.5 ± 0.4°C in the absence of inhibitor to 48.9 ± 0.2, 49.0 ± 0.0, 49.8 ± 0.2°C in the presence of 0.5, 1.0, and 2 mM vaborbactam, respectively (Fig 3). This modest protein stabilization contrasts to the much larger stabilization as a result of either ceftazidime, cefoperazone (Fig 1), or azlocillin binding to *P*. *aeruginosa* PBP3, which yielded increases in *T*_*m*_ in the 10–15°C range [31, 42]. However, it is not required for potent PBP3 inhibitors to stabilize PBP3 upon binding as the carbapenems meropenem and imipenem destabilized PBP3 upon binding; conformational changes and ability to displace unstable water molecules were postulated as contributing factors [30]. Note that a simple correlation between change in protein stability upon ligand binding and ligand binding affinity to the same protein is not evident [43]. Both the Bocillin™ and DSF results indicate that vaborbactam binds PBP3, which compelled us to analyze this complex crystallographically.

### Crystallographic analysis vaborbactam binding to PBP3

**Fig 4:**
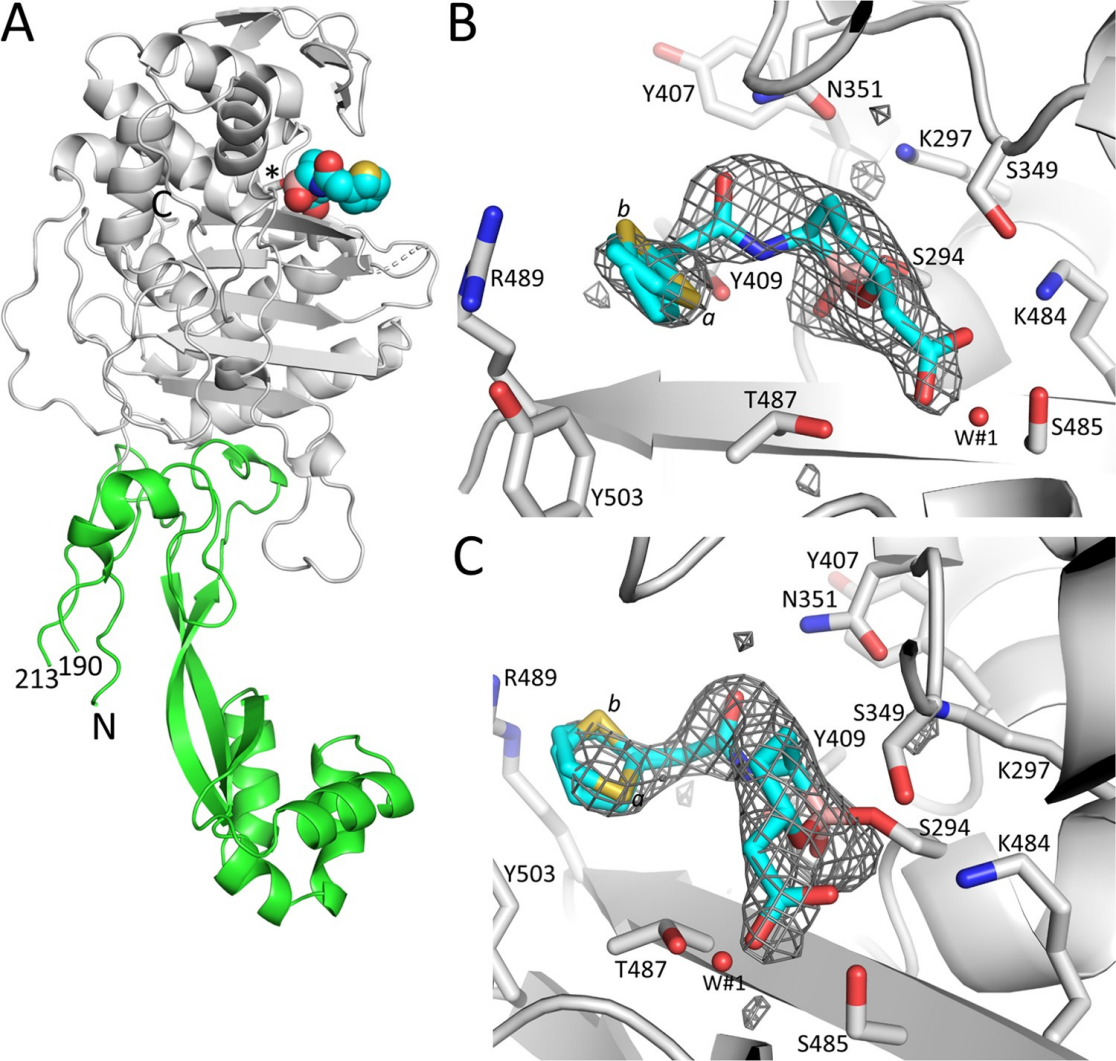
Structure and electron density of vaborbactam bound covalently in the active site of *P*. *aeruginosa* PBP3. A, simplified diagram of the crystal structure of PBP3 complexed to vaborbactam. The catalytic domain is colored grey; the N-terminal domain is in green. Vaborbactam is depicted in spheres with the carbon atoms in cyan. The catalytic S294 to which vaborbactam is covalently bonded is shown in stick model (labeled ‘*’). The N- and C-termini are labeled, and the residue numbers at the ends of the large missing region in the N-terminal domain are labeled. B, Unbiased omit *Fo-Fc* difference density is obtained after removing vaborbactam from the model and subsequently performing 10 cycles of crystallographic refinement before map calculation. Vaborbactam is shown in a stick model with cyan carbon atoms. The difference density is contoured at the 2.75 σ level. C, same as B, but the view is rotated about 90°.

The crystal structure of *P*. *aeruginosa* PBP3 with covalently bound vaborbactam is refined to 2.2 Å resolution (see Table 1 for additional refinement statistics). The PBP3 structure contains a non-catalytic N-terminal domain and the C-terminal catalytic domain (Fig 4A). The cyclic boronic acid vaborbactam is bound in the active site in the same location as β-lactams such as ceftazidime and meropenem inhibiting this PBP3. Unbiased active site electron density shows vaborbactam covalently attached to the essential S294 in the catalytic domain (Fig 4B and 4C); the boron bound to S294 is in *sp*^*3*^ configuration similar as observed when vaborbactam binds to serine β-lactamases. Electron density is present for the cyclic boronate ring and the amide moiety, the carboxyl group, and the thiophene ring of vaborbactam are readily seen. The latter density is weaker, with the thiophene ring moiety refined in two conformations (0.7 and 0.3 occupancies, labeled *a* and *b*, respectively). The overall weaker density for this thiophene ring is likely a result of multiple conformations/disorder in this moiety.

**Fig 5:**
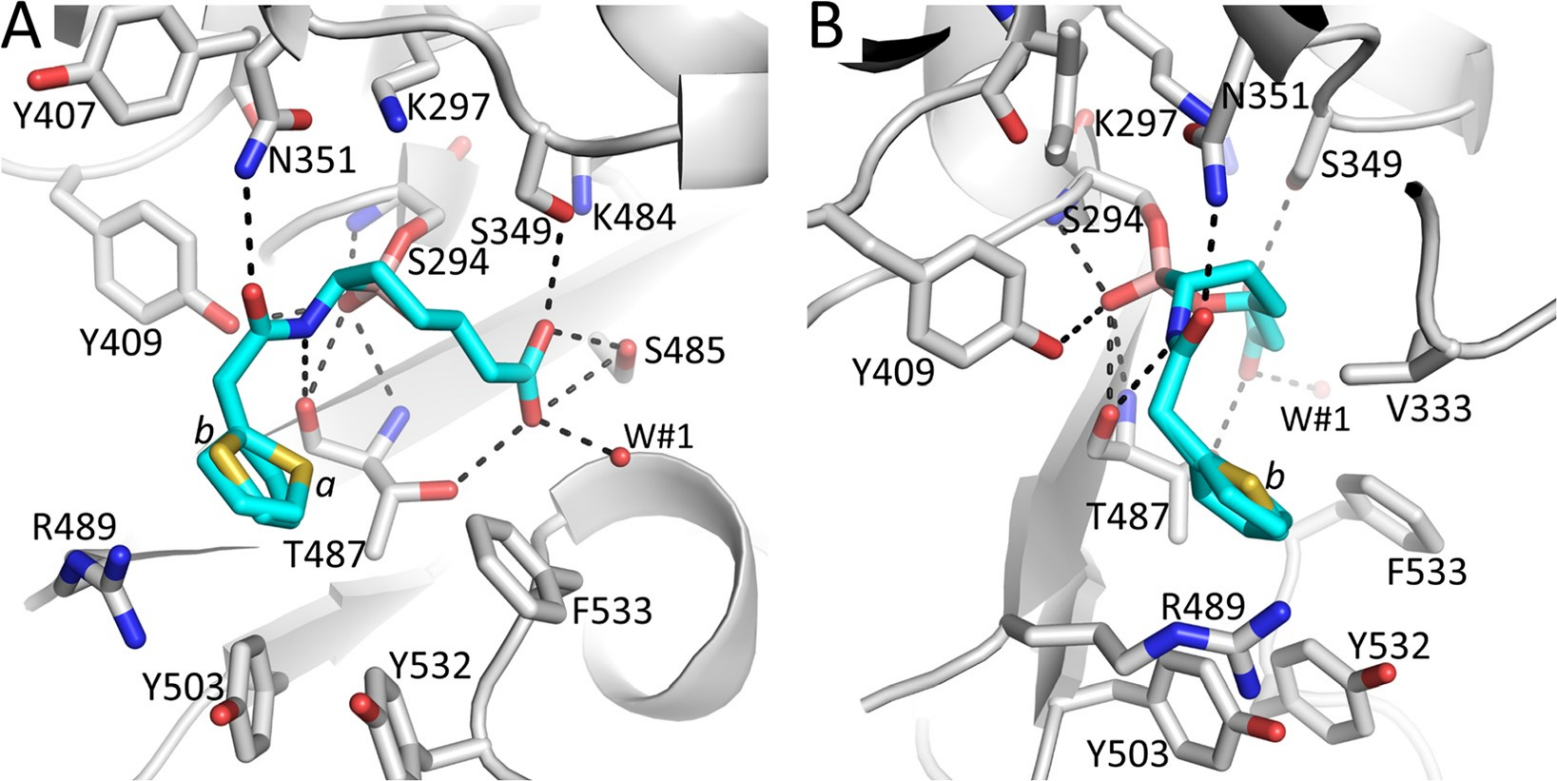
Figure showing vaborbactam binding in the active site of *P*. *aeruginosa* PBP3. A, Close-up view of vaborbactam in the PBP3 active site. Hydrogen bonds are depicted as dashed lines. Atom coloring is as in Fig 4. The two conformations of the aminothiazole containing moiety of vaborbactam are indicated by labels ‘a’ and ‘b’ near their respective sulfur atoms. A crystallographically observed water molecule interacting with vaborbactam is shown as a red sphere (labeled W#1). B, same as A but view is rotated about 90°.

Vaborbactam makes several important interactions in the PBP3 active site. The boron atom makes a covalent (dative) bond with the oxygen of the catalytic S294 (Fig 5). The boron, upon bonding to S294 changed its hybridization from *sp*^*2*^ to *sp*^*3*^. The hydroxyl moiety of the boronic acid is situated in the oxyanion hole. This hydroxyl moiety creates hydrogen bonding interactions with the backbone nitrogens of S294 and T487, with the hydroxyl of Y409, and with the backbone oxygen of T487 (Fig 5). The amide oxygen of vaborbactam forms a hydrogen bond with the side chain nitrogen of N351; the amide nitrogen is hydrogen bonding with the backbone oxygen of T487 (Fig 5). The carboxyl group of vaborbactam forms hydrogen bonds with T487, S485, and S349 and with water molecule W#1 (Fig 5). The thiophene ring is seen forming hydrophobic interactions (< 4Å) with V333, Y503, and the T487 side chain (Fig 5). The carbon atoms of the boronate ring establish hydrophobic interactions with V333 and the Cβ atom of S349.

### Structural comparisons to other PBP3 complexes

The vaborbactam PBP3 complex structure allows comparison with other PBP3 complexes bound with PBP3-targeting β-lactam antibiotics including ceftazidime, cefoperazone, meropenem, and the monobactam aztreonam [30, 38, 42]. Such analysis could provide insights into the characteristics of potent inhibitors binding to PBP3 compared to vaborbactam, which has a new (boronic acid-based) PBP-inhibiting scaffold and has relatively less affinity for PBP3. In our comparisons, we are also including an additional new PBP-inhibiting scaffold, that of the DBO zidebactam, the structure of which was recently determined in complex with PBP3 [18].

**Fig 6:**
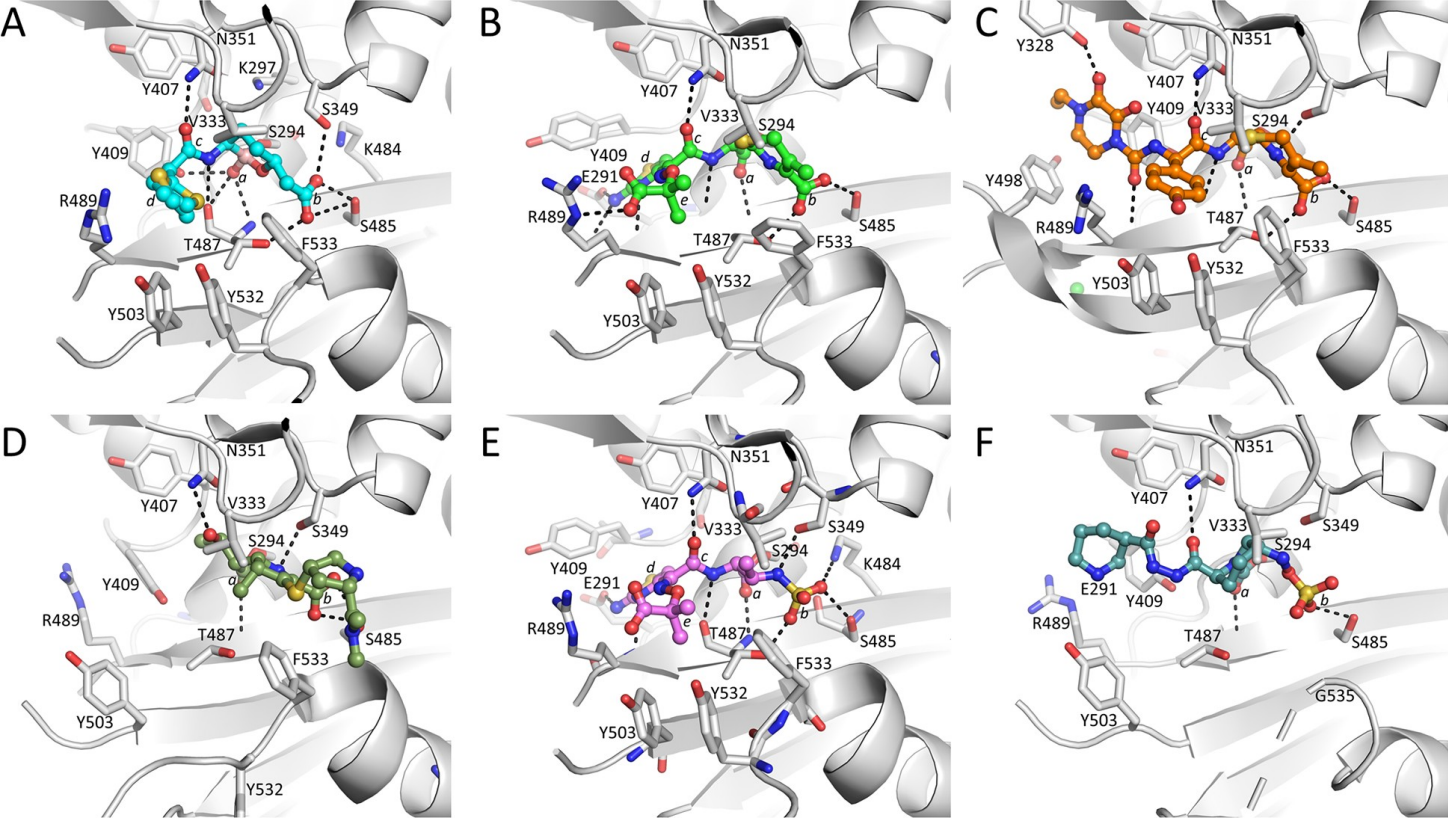
Comparisons of vaborbactam-bound PBP3 with the *P*. *aeruginosa* PBP3-β-lactam, monobactam, and DBO complexes. A, vaborbactam-bound PBP3 structure with ligand in ball-and-stick representation with cyan-colored carbon atoms. The ligand’s oxygen located in the oxyanion hole (*a*), the carboxyl (*b*), the amide (*c*), and the five-membered thiophene (*d*) are labeled. B, ceftazidime-bound PBP3 (PDBid 3PBO, [30]; ceftazidime is shown with green-colored carbon atoms). The PBP3 superimposition with the vaborbactam PBP3 structure resulted in a RMSD of 0.45 Å for 473 Cα atoms. In addition to the same moieties *a*-*c* as in A, also labeled are the five-membered aminothiazole ring (*d*), and ceftazidime’s 2-carboxypropan-2-yl group (*e*). C, cefoperazone-bound PBP3 (PDBid 5DF8, [42]; cefoperazone is shown with gold-colored carbon atoms). The PBP3 superimposition with the vaborbactam PBP3 structure resulted in a RMSD of 1.76 Å for 473 Cα atoms. Moieties, if present as in A, are labeled. D, meropenem-bound PBP3 (PDBid 3PBR, [30]; meropenem is shown with dark green-colored carbon atoms). The PBP3 superimposition with the vaborbactam PBP3 structure resulted in a RMSD of 0.78 Å for 473 Cα atoms. Moieties, if present as in A, are labeled. E, aztreonam-bound PBP3 (PDBid 3PBS, [30]; aztreonam is shown with pink-colored carbon atoms). The PBP3 superimposition with the vaborbactam PBP3 structure resulted in a RMSD of 0.66 Å for 473 Cα atoms. Moieties, if present as in B, are labeled with *b* now indicating the sulfo moiety. F, zidebactam-bound PBP3 (PDBid 7KIW, [18]; zidebactam is shown with teal-colored carbon atoms). Moieties, if present as in E, are labeled.

Structural comparisons of the PBP3 complexes of vaborbactam and zidebactam, and PBP3 inhibitors ceftazidime, cefoperazone, meropenem, and aztreonam reveal similarities and differences in their binding mode and active site conformations (Figs 1 and 6 and S1 Fig). Firstly, all inhibitors have a similar orientation in that they are covalently bound to S294, and their scaffold carboxyl (or sulfo) moiety hydrogen bonds with S485 and often also with T487 (Fig 6). Secondly, all inhibitors position an oxygen atom in the oxyanion hole (formed by backbone nitrogens of T487 and S294). Thirdly, the inhibitors make a hydrogen bond to the nitrogen of the side chain of N351 via an oxygen atom; if this oxygen belongs to an amide moiety in the inhibitor, then an additional hydrogen bond is formed via this amide nitrogen with the backbone oxygen of T487 (Fig 6). Furthermore, for inhibitors with an aminothiazole ring, such as ceftazidime and aztreonam, the side chain of Y409 has rotated away to generate a “pocket” for this aminothiazole ring; this ring makes several hydrogen bonds when bound in this conformation. Finally, ceftazidime, aztreonam, and cefoperazone binding cause residues Y532 and F533 to be ordered and, together with the shifted Y503, form the “aromatic wall” in the PBP3 active site [30, 42]. Meropenem binding leads to a similar loop shift involving F533, yet Y532 points away from the active site, likely due to meropenem not having a large R_1_ group (Figs 1 and 6).

Residue F533 is important for β-lactam inhibition of PBP3 since the F533L substitution results in resistance to some β-lactams, including ceftazidime and meropenem [44]. Additionally, residues F533 and V333 form the hydrophobic bridge that partially covers the bound inhibitor in these β-lactam and monobactam structures (Fig 6). The aromatic wall and hydrophobic bridge also formed in other PBP3 complexes, such as with the γ-lactams YU253911 and YU253434 [19, 45]. In apo PBP3 structures, this loop is more distant from the active site [30, 46]. Although both the aromatic wall (residues Y503, Y532, and F533) and hydrophobic bridge (V333 and F533) are formed upon vaborbactam binding (Fig 6), the crystallographically refined temperature factors of these residues are considerably higher compared to the core of the catalytic domain (S1 Fig). This contrasts with the ceftazidime, cefoperazone, meropenem, and aztreonam PBP3 complexes, where the aromatic wall and hydrophobic bridge residues have much lower temperature factors indicating a more ordered structure (S1 Fig). Like vaborbactam, zidebactam is also a relatively less potent PBP3 inhibitor, and its binding to PBP3 led to neither the formation of the aromatic wall nor hydrophobic bridge (Fig 6 and S1 Fig) [18]. Based upon these observations, we posit that the more mobile aromatic wall and hydrophobic bridge residues in the vaborbactam and zidebactam complexes are due to the presence of fewer hydrophobic moieties in these low affinity PBP3 inhibitors that could drive such stabilizing conformational changes (both inhibitors lack an R_2_ group). Furthermore, unlike the other five inhibitor complexes in Fig 6, zidebactam binding did not induce a shift in the β3 strand (containing T487) as the active site in the zidebactam complex was observed to harbor an apo-PBP3-like conformation [18]. Although zidebactam can bind PBP3, it prefers to inhibit PBP2 through which it mediates its “enhancer” properties [13, 15, 18]. Very recently, another group has also determined the structure of *P*. *aeruginosa* PBP3 bound to vaborbactam ([47]; PDB id 7AUH). The vaborbactam PBP3 interactions in this analysis show only a single conformation of the aminothiazole ring (inspection of the temperature factors of the 7AUH coordinates also revealed the same trend as we have described).

### Structural comparison of vaborbactam binding to KPC-2 β-lactamase

**Fig 7:**
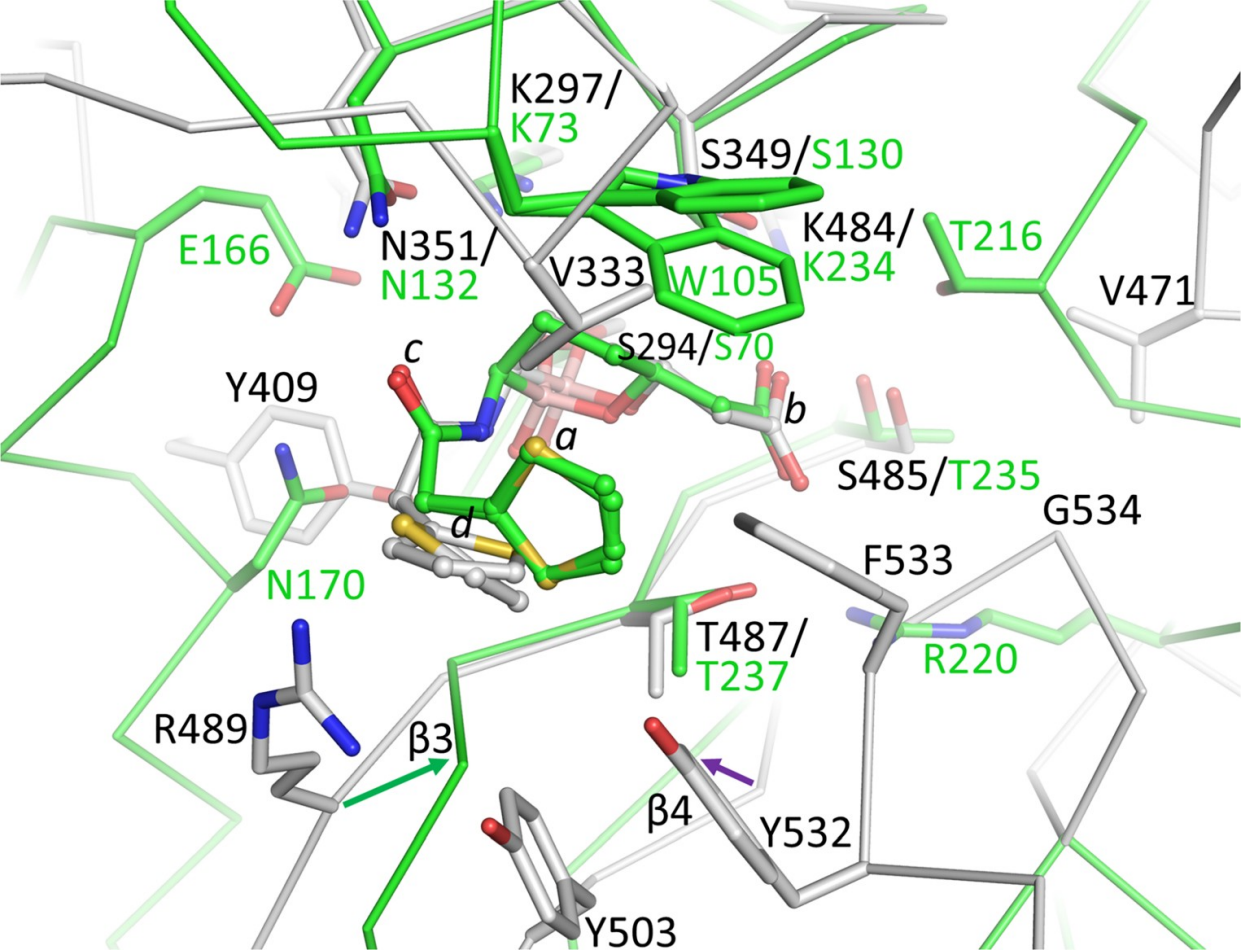
Superimposition of vaborbactam-bound PBP3 onto KPC-2 β-lactamase bound vaborbactam structure. The colors and representation of PBP3 and its bound vaborbactam are as in Fig 6A. KPC-2 (PDBid 6V7I is shown in green backbone trace and green carbon atoms; its bound vaborbactam is depicted in ball-and-stick with green carbon atoms. Shifts in the β3 and β4 strands are indicated by arrows. The active site Cα atoms of PBP3 residues 294–301, 346–353, 481–486, and 505–509, were superimpositioned onto their equivalent atoms in KPC-2 (residues 70–77, 127–134, 231–236, and 244–248) yielding an RMSD of 0.97 Å for 27 Cα atoms.

The crystal structure of PBP3 bound to vaborbactam allows a comparison as to how vaborbactam inhibits the β-lactamase KPC-2 ([48]; PDB ID 6V7I), one of the intended targets of this β-lactamase inhibitor (*K*_d_ of vaborbactam for KPC-2 is 7.4 nM [48]). Superimposition of these two structures, which are evolutionarily related, reveals that vaborbactam is covalently bound in a similar orientation with similar interactions (Fig 7). The catalytic S70, to which vaborbactam is covalently attached, K73, and K234 are equivalent to PBP3 residues S294, K297, and K484, respectively (Fig 7). The carbonyl oxygen of vaborbactam is in the oxyanion hole in both structures, and the boronate ring and attached carboxyl moiety are in a similar position and orientation. The carboxyl moiety makes hydrogen bonds with KPC-2 residues T235, T237, and S130 (Fig 7); these interactions are equivalent in the vaborbactam PBP3 structure with corresponding residues S485, T487, and S349, respectively. Likewise, the amide moiety of vaborbactam makes identical hydrogen bonds in the KPC-2 and PBP3 active sites (Fig 7). An additional similarity is that the aminothiazole moiety in both vaborbactam-bound structures adopts two conformations (Fig 7). One important difference is that the KPC-2 active site residue, W105, provides hydrophobic interactions with vaborbactam; PBP3 residue V333 provides analogous interactions in the corresponding part of the active site in PBP3, although likely much less extensive because of its smaller size compared to W105 of KPC-2. Also, PBP3 possesses residue Y409 that makes a hydrogen bond with the boronate oxygen, whereas KPC-2 positions N170 at this position which makes van der Waals interactions with the amide moiety of vaborbactam. An additional difference is that KPC-2 has the R220 residue positioned at 3.7 Å from the carboxyl moiety of vaborbactam, providing additional electrostatic interactions with this ligand moiety; PBP3 does not have an analogous residue situated nearby (Fig 7). Also, differences in the position of parts of the β3 strand where PBP3 residue R489 is situated (Fig 7) as well as some minor shifts in parts of the β4 strand are also evident. Note that the root-mean-square-deviation (RMSD) for superimposing 27 Cα atoms in the active site was 0.97 Å indicating that these residues are positioned similarly, but still with significant variation in their position, which could also contribute to the overall weaker affinity of vaborbactam for PBP3 compared to KPC-2. Overall, we hypothesize that the diminished affinity of vaborbactam for PBP3 is due to the above-observed differences between PBP3 and KPC-2 and that vaborbactam binding to PBP3 is accompanied by a conformational change involving residues Y532 and Y533 that is not needed in KPC-2.

### Molecular Dynamics (MD) simulations of vaborbactam bound to PBP3

To complement the crystal structure and gain insights into the dynamics of active site residues and active-site loops interacting with vaborbactam when bound to PBP3, we carried out MD simulations. The protonation state of K297, part of the conserved SXXK PBP/β-lactamase motif, was set as protonated based on the observed protonation state of the corresponding lysine protonation state in the 1.00 Å resolution KPC-2 vaborbactam crystal structure [49] and the structure of a related Class A β-lactamase, the CTX-M-15 vaborbactam structure also determined at 1.0 Å resolution (these high-resolution structures allow visualization of hydrogen atoms) [48]. We also carried out a second simulation with this K297 set as neutral for comparison.

**Fig 8:**
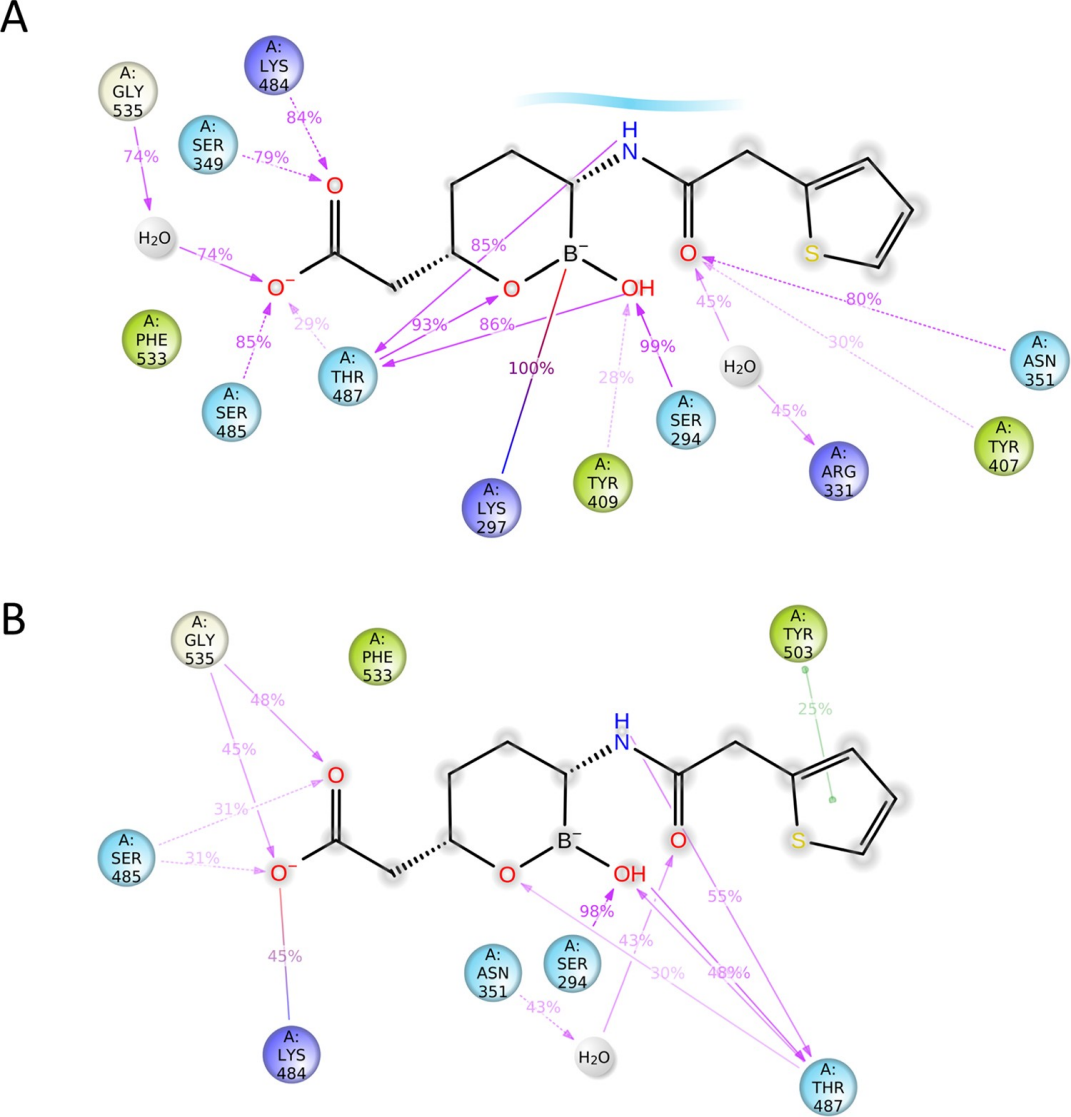
Vaborbactam interactions with the active site of PBP3 during MD simulations. A, Vaborbactam PBP3 active site interactions during MD simulations with K297 in the protonated state analyzed using the Schrodinger Simulation Interactions Diagram program. Hydrogen bonds with side chains (pink dashed arrow) and main chains (pink solid arrow), and salt bridge interactions (purple-pink line) are indicated, and their percentage present during the simulation is shown. B, same as A but with K297 kept neutral during the simulation.

The 100 ns simulation of PBP3 with vaborbactam yielded a stable protein structure with a RMSD for Cα atoms of around 3.7 Å for this multidomain protein (S2A Fig). The root-mean-square-fluctuations (RMSF) per residue during the simulation follow a similar trend as the crystallographically refined temperature factors (S2B and S2C Fig). The simulation showed that the covalently bound vaborbactam made similar hydrogen bond interactions (Fig 8) as were crystallographically observed (Fig 5). These include stable interactions of the boron hydroxyl in the oxyanion hole formed by the backbone atoms of residues S294 and T487 as well as, to a lesser degree, with the hydroxyl of Y409 (Fig 8 and S2 Fig). The amide moiety of vaborbactam forms a stable hydrogen bond with N351 and the backbone oxygen of T487 in the simulation, as was also observed in the crystal structure. During the simulation, the carboxyl group makes hydrogen bonds with S485, S349, K484, and to a lesser degree with T487; these interactions are also largely in agreement with the crystal structure (the distance of the carboxyl oxygen atom of vaborbactam with K484 in the crystal structure is 3.5 Å).

When we investigated hydrophobic ligand interactions, the simulation also showed that residues Y503, Y532, and Y533 do not form stable van der Waals interactions with vaborbactam (S2D Fig) which is in concordance with their observed higher temperature factors of the crystal structure (S1 Fig). Also, the aminothiazole ring was observed in two conformations (S2E Fig) as was also observed crystallographically (Fig 5).

One notable difference between the MD simulation ligand interactions and the crystal structure is that the main chain nitrogen of T487 makes a direct hydrogen bond with the oxygen in the boronate ring (S1 Fig); in the crystal structure, this is a 3.3 Å distance which is almost within hydrogen-bonding range (Fig 5). Perhaps, this can be explained by the difference in the temperature of the simulation, 310 K, compared to the 100 K temperature of the frozen crystal during the diffraction data collection. Interestingly, when the simulation was carried out with a neutral K297 residue, the ligand amide oxygen interactions with the side chain of N351 were not observed (Fig 8B). Furthermore, the hydrogen bond interactions with the carboxyl oxygens of vaborbactam were occurring much less frequently; a similar and also less frequent interaction was observed for the amide nitrogen atom of vaborbactam (Fig 8B). Comparison of the protonated and neutral K297 MD simulations suggests that the K297 is indeed protonated with the vaborbactam protein interactions more closely resembling the crystal structure. K297 in the crystal structure is involved in an intricate hydrogen bonding network as it donates three hydrogen bonds: with the oxygen of the N351 side chain, the backbone oxygen of S349, and the Oγ atom of the catalytic S294. Only a protonated K297 would be able to make these three stabilizing interactions. The 2.6 Å hydrogen bond of a K297 with the oxygen atom of the N351 side chain likely keeps N351 oriented such that the side chain nitrogen atom can provide a stable interaction with the amide oxygen of vaborbactam (Fig 5). Furthermore, the interaction diagram in Fig 8 also showed the presence of a salt bridge between K297 and the negatively charged *sp*^*3*^ boron atom; this distance in the crystal structure is 4.2 Å which would also represent a (buried) salt-bridge interaction.

In summary, we show that the β-lactamase inhibitor vaborbactam can bind and inhibit the evolutionarily related *P*. *aeruginosa* PBP3 but with a much higher IC_50_ and thus less affinity compared to β-lactams. The crystal structure of vaborbactam bound to PBP3 demonstrates a covalently bound vaborbactam complex with the ligand making a number of hydrogen bonding and van der Waals interactions. Most of the hydrogen bonding interactions of vaborbactam in the active site are similar to those observed for β-lactam binding to PBP3. Although vaborbactam binding did lead to the formation of the active site’s aromatic wall and hydrophobic bridge, these regions are more mobile in the crystal structure which we hypothesize is due to the more limited van der Waals interactions that vaborbactam makes with these active site regions (possibly in part due its smaller R_1_ group and lack of a R_2_ group). Despite these caveats, our studies show the characterization of PBP inhibition by a cyclic boronate, and the results presented herein establish a path forward to the development of more potent boronic-acid-based non-β-lactam PBP-targeting antibiotics.

## Supporting information

S1 Fig Temperature factor comparison of the PBP3 vaborbactam complex and β-lactam, monobactam, and DBO PBP3 complexes. A-F, the view, and orientation are the same as in Fig 6. The temperature factor of each atom is color ramped in Pymol (spectrum b blue_white_red minimum = 30, maximum = 80).
(TIF)

S2 Fig Molecular dynamics simulation of the P. aeruginosa PBP3 vaborbactam complex analyzed using the Schrodinger simulation interactions diagram program. A, RMSD of Cα atoms (blue) and ligand atoms (red) during the 100 ns simulation with K297 of PBP3 protonated. B, RMSF of each residue during the simulation. C, crystallographically refined temperature factors of the main chain plotted per residue. D, histogram of protein-ligand interactions with hydrogen bond, hydrophobic, salt bridge interactions, and water-mediated interactions shown in green, purple, red, and blue, respectively. Some residues make two or more interactions hence their fraction being larger than 1.0. E, vaborbactam torsion angle distributions during the simulation.
(TIF)

S1 Raw images Original entire fluorescence scan image for Fig 2. (PDF)

S1 File PDB coordinates validation report. (PDF)
