# Supplementary figures and images for: Structural analysis of the boronic acid β-lactamase inhibitor vaborbactam binding to Pseudomonas aeruginosa penicillin-binding protein 3
Source: PLoS One. 2021 Oct 15;16(10):e0258359. doi: 10.1371/journal.pone.0258359 (PMC8519428; doi:10.1371/journal.pone.0258359)

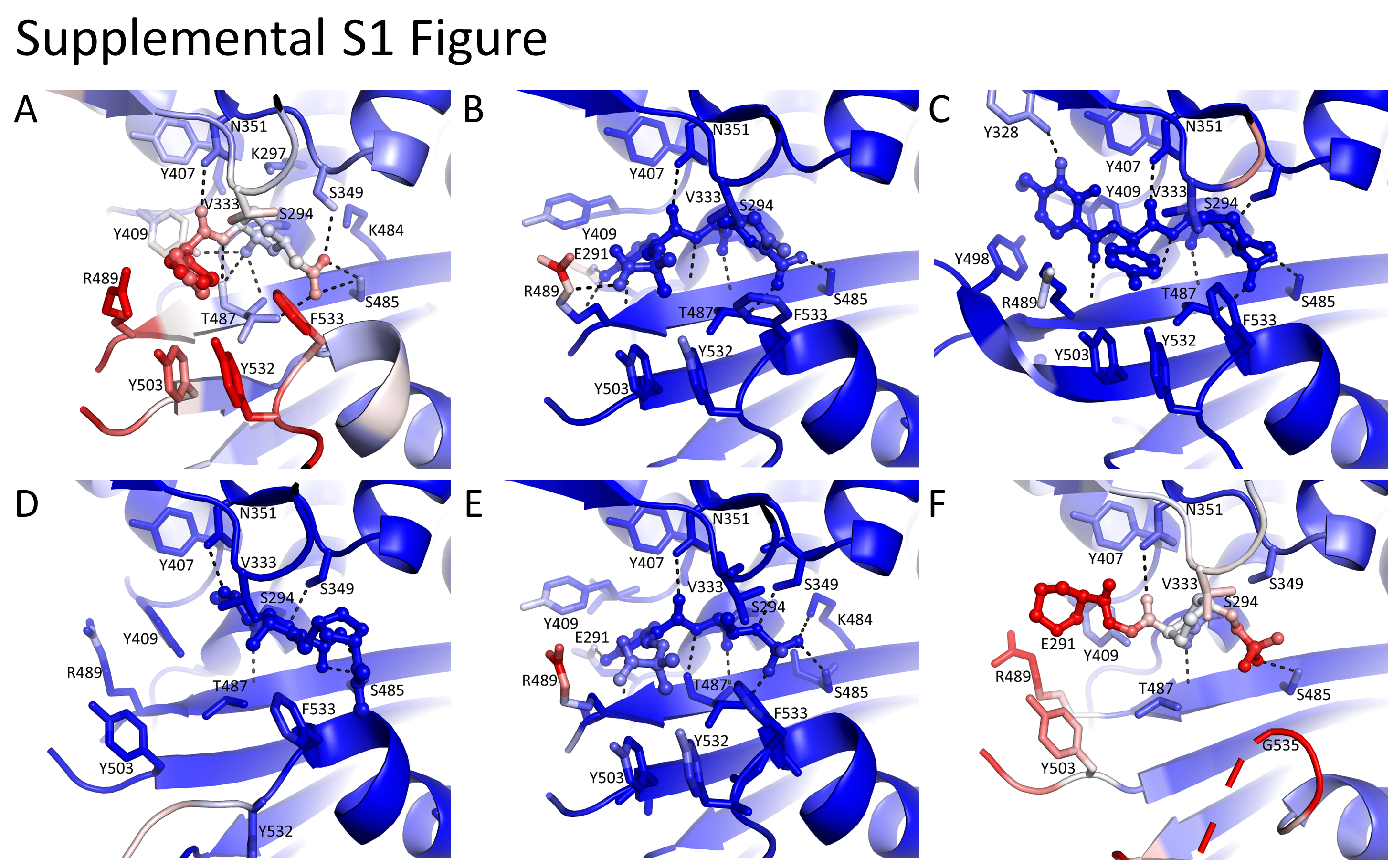

Supplement: S1 Fig — A-F, the view, and orientation are the same as in Fig 6. The temperature factor of each atom is color ramped in Pymol (spectrum b blue_white_red minimum = 30, maximum = 80). (TIF) [file pone.0258359.s001.tif]

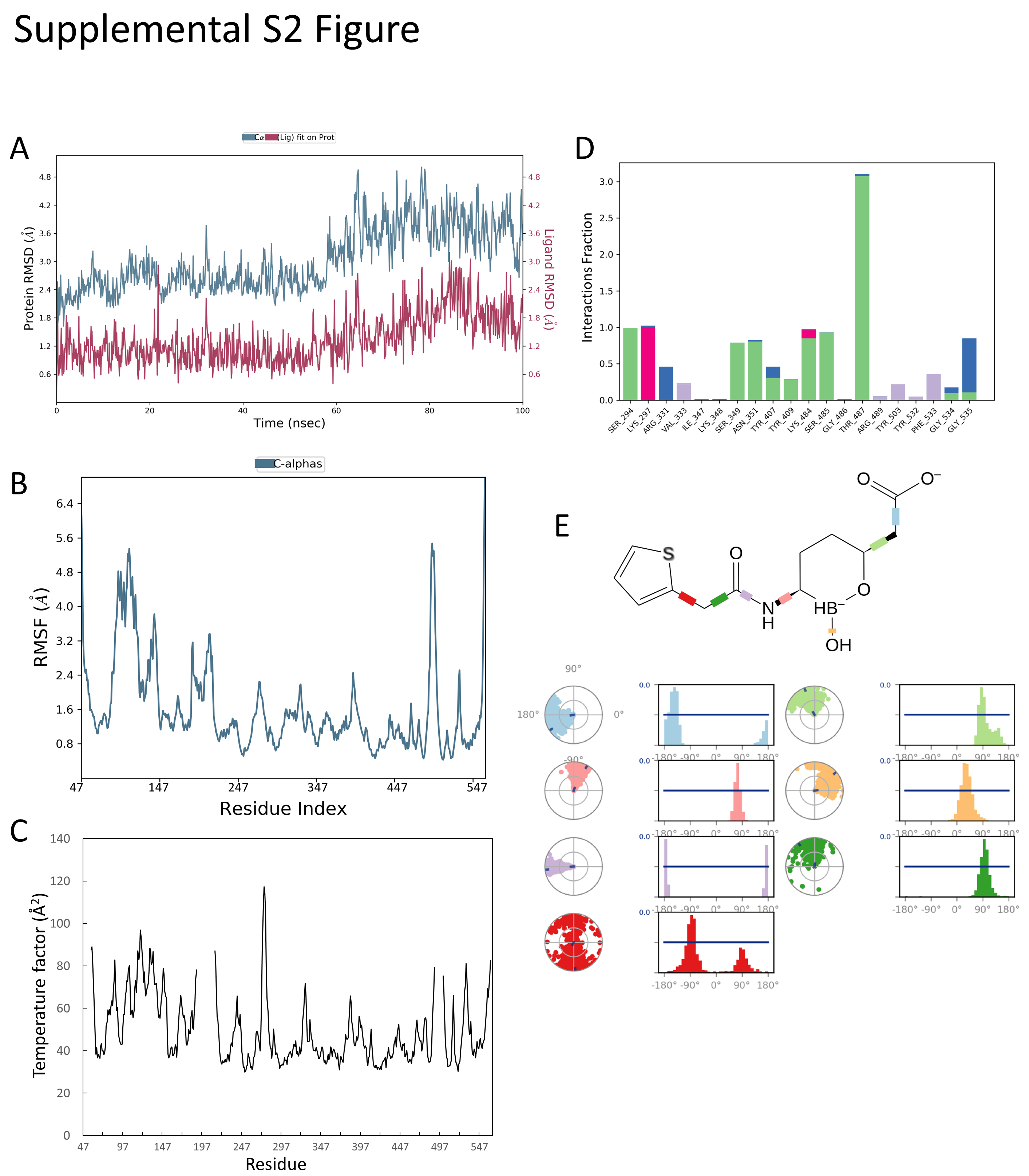

Supplement: S2 Fig — A, RMSD of Cα atoms (blue) and ligand atoms (red) during the 100 ns simulation with K297 of PBP3 protonated. B, RMSF of each residue during the simulation. C, crystallographically refined temperature factors of the main chain plotted per residue. D, histogram of protein-ligand interactions with hydrogen bond, hydrophobic, salt bridge interactions, and water-mediated interactions shown in green, purple, red, and blue, respectively. Some residues make two or more interactions hence their fraction being larger than 1.0. E, vaborbactam torsion angle distributions during the simulation. (TIF) [file pone.0258359.s002.tif]
