# Supplementary material for: Structural analysis of the boronic acid β-lactamase inhibitor vaborbactam binding to Pseudomonas aeruginosa penicillin-binding protein 3
Source: PLoS One. 2021 Oct 15;16(10):e0258359. doi: 10.1371/journal.pone.0258359 (PMC8519428; doi:10.1371/journal.pone.0258359)

Entire fluorescence image of Fig 2

Image taken using Bio-rad ChemiDoc MP Imaging System using Alexa 488 fluorescent channel (2.5 sec exposure). SDS PAGE gel used: Any kD™ Mini-PROTEAN® TGX™ Precast Protein Gel

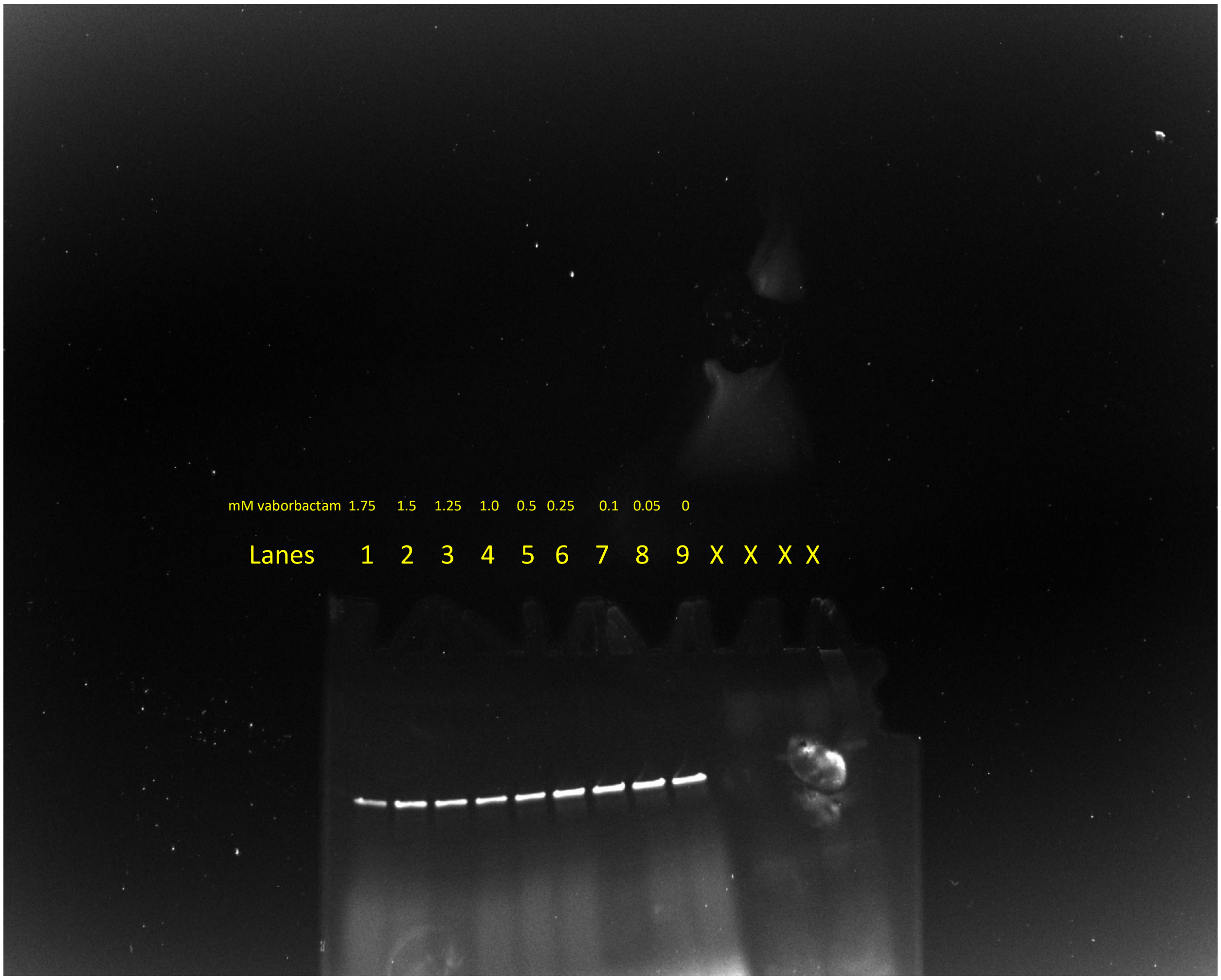

Supplement: S1 Raw images — (PDF) [file pone.0258359.s003.pdf]
